# Comparative Transcriptome Analysis Identifies Genes Involved in Diosgenin Biosynthesis in *Trigonella foenum-graecum* L.

**DOI:** 10.3390/molecules24010140

**Published:** 2019-01-01

**Authors:** Chen Zhou, Xiaohua Li, Zilin Zhou, Changfu Li, Yansheng Zhang

**Affiliations:** 1 Key Laboratory of Plant Germplasm Enhancement and Specialty Agriculture, Wuhan Botanical Garden, Chinese Academy of Sciences, Wuhan 430074, China; zhouchen179@sina.com (C.Z.); lixiaohua14@mails.ucas.edu.cn (X.L.); zhouzilin16@mails.ucas.ac.cn (Z.Z.); lichangfu@wbgcas.cn (C.L.); 2 University of Chinese Academy of Sciences, Beijing 100049, China; 3 Shanghai Key Laboratory of Bio-Energy Crops, Research Center for Natural Products, School of Life Sciences, Shanghai University, Shanghai 200444, China

**Keywords:** *Trigonella foenum-graecum* L., diosgenin biosynthesis, glycosyltransferase, cytochrome P450, transcriptional factors

## Abstract

*Trigonella foenum-graecum* L. (fenugreek) is a valuable resource of producing diosgenin which serves as a substrate for synthesizing more than two hundred kinds of steroidal drugs. Phytochemical analysis indicated that methyl jasmonate (MeJA) efficiently induced diosgenin biosynthesis in fenugreek seedlings. Though early steps up to cholesterol have recently been elucidated in plants, cytochrome P450 (CYP)- and glycosyltransferase (GT)-encoding genes involved in the late steps from cholesterol to diosgenin remain unknown. This study established comparative fenugreek transcriptome datasets from the MeJA-treated seedlings and the corresponding control lines. Differential gene expression analysis identified a number of MeJA-induced CYP and GT candidate genes. Further gene expression pattern analysis across a different MeJA-treating time points, together with a phylogenetic analysis, suggested specific family members of CYPs and GTs that may participate in the late steps during diosgenin biosynthesis. MeJA-induced transcription factors (TFs) that may play regulatory roles in diosgenin biosynthesis were also discussed. This study provided a valuable genetic resource to functionally characterize the genes involved in diosgenin biosynthesis, which will push forward the production of diosgenin in microbial organisms using a promising synthetic biology strategy.

## 1. Introduction

*Trigonella foenum-graecum* L. (fenugreek) is an annual dicotyledonous legume belonging to family *Fabaceae* (subfamily Papilionaceae), which is widely distributed throughout the world [1,2]. Fenugreek has reportedly been used for the treatment of poor digestion, eczema, inflammation, anemia and bronchitis [3,4,5]. Studies showed that fenugreek contains diverse bioactive compounds with anticancer, antidiabetic, and antimicrobial activities, and cholesterol-lowering effects [6,7,8]. Among these bioactive chemicals, dioscin, a steroidal saponin, has received the most interest due to its antitumor, anticancer and anti-inflammatory activities [9,10,11]. Moreover, the aglycone part of dioscin, named diosgenin, is an important precursor for synthesizing more than two hundred steroidal drugs such as contraceptives, testosterone, progesterone and glucocorticoids [7,12].

**Scheme 1:**
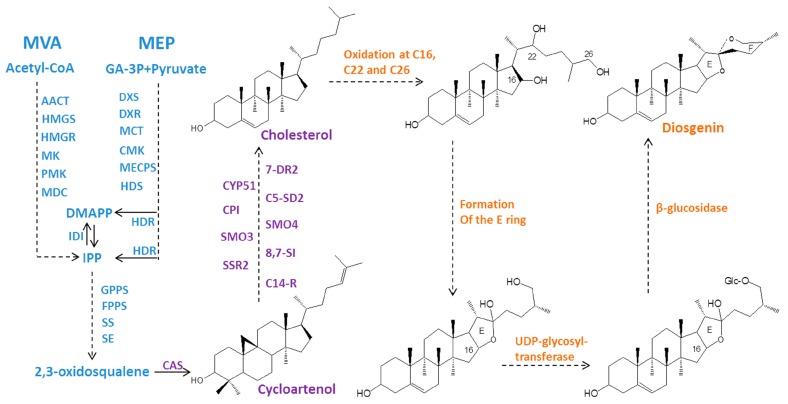
Proposed diosgenin biosynthetic pathway. Abbreviations: GA-3P, d-Glyceraldehyde 3-phosphate; DXP: 1-Deoxy-d-xylulose-5-phosphate; MEP, 2-C-Methyl-d-erythritol-4-phoshate; CDP-ME, 4-(Cytidine 5′-diphospho)-2-C-methyl-d-erythritol; CDP-MEP, 2-Phospho-4-(cytidine 5′-diphospho)-2-C-methyl-d-erythritol; ME-cPP, 2-C-Methyl-d-erythritol-2,4-cyclodiphosphate; HMBPP, 1-Hydroxy-2-methyl-2-butenyl 4-diphosphate; DMAPP, Dimethylallyl diphosphate; IPP, Isopentenyl diphosphate; GPP, Geranyl diphosphate; FPP, Farnesyl diphosphate; UGT: UDP- glycosyltransferases; AACT, acetyl CoA acetyltransferase; HMGS, hydroxymethylglutaryl CoA synthase; HMGR, 3-hydroxy-3-methylglutaryl CoA reductase; MK, mevalonate kinase; PMK, phosphomevalonate kinase; MDC, mevalonate diphosphosphate decarboxylase; DXS, 1-deoxy-d-xylulose-5-phosphate synthase; DXR,1-deoxy-d-xylulose-5-phosphate reductoisomerase; MCT, 2-C-methyl-d-erythritol 4-phosphatecytidylyl transferase; CMK, 4-diphosphocytidyl-2-C- methyl-d-erythritol kinase; MECPS,2-C-methyl-d-erythritol-2, 4-cyclodiphosphate synthase; HDS, 4-hydroxy-3-methylbut-2-(*E*)-enyldiphosphate synthase; HDR, 4-hydroxy-3-methylbut-2-enyl- diphosphate reductase; IPI, IPP diphosphate isomerase; GPPS, geranyldiphosphate synthase; FPPS, farnesyl diphosphate synthase; SS, squalene synthase; SE, squalene epoxidase; CAS, cycloartenol synthase; SSR, sterol side chain reductase; SMO, sterol 4a–methyloxidase; CPI, cycloeucalenol cycloisomerase; CYP51, sterol C-14demethylase; C14-R, sterol C-14 reductase; 8,7-SI, sterol 8,7-isomerase; C5-SD, sterol C-5 (6) desaturase; 7-DR, 7-dihydrocholesterol reductase.

At present, the direct solvent extraction of plant materials, which consumes tons of plant materials per year and brings serious environmental pollution, is the only approach to obtain diosgenin. The emergence of synthetic biology provides an ideal approach to produce diosgenin but with the condition that a complete list of diosgenin biosynthesis genes be available. However, despite the pharmacological importance of diosgenin, its biosynthesis pathway remains not completely understood, and especially knowledge of its downstream pathway genes is still lacking. Several research groups have previously proposed that diosgenin biosynthesis could be divided into three stages [13,14,15] (Scheme 1). 

The first stage leads to the formation of 2, 3-oxidosqualene through isoprenoid units either derived from the cytosolic mevalonate (MVA) or derived from the plastid targeted 2-C-methyl-d-erythrirtol-4-phosphate (MEP) pathway. Genes in the first stage have been extensively investigated [16,17]. The second stage includes ten enzymatic steps to convert 2, 3-oxidosqualene into cholesterol, and they have recently been characterized at the molecular level in plants [18]. However, both the pathway genes and enzymes involved in the last stage from cholesterol to diosgenin remain largely unknown, which severely hinders the use of synthetic biology to produce diosgenin in microorganisms or other biological systems. In view of the molecular structure of diosgenin, multiple oxidations at the C-22, C-16 and C-26 positions of cholesterol are required [14], which are possibly mediated by specific cytochrome P450s (CYPs). Based on the previous reports [13,19,20], in addition to the sequential oxidation reactions, the addition of glucosyl moiety to the C-26 hydroxyl group followed by the cleavage of this glucosyl bond would be needed to form the F ring of diosgenin (Scheme 1). Thus, a UDP-glucosyltransferase (UGT) in catalyzing the glucosyl transfer reaction and a β-glucosidase in removing the glucosyl unit might be involved in disosgenin biosynthesis as well (Scheme 1).

Comparative transcriptome analysis has accelerated the discovery of genes involved in the pathways of several important secondary metabolites [21,22,23]. Here, a comparatively transcriptomic analysis was applied to MeJA-treated and the control fenugreek seedlings, as an obviously MeJA-induced improvement in diosgenin biosynthesis was observed in this study, supporting previous reports [24]. Through differential expression analysis, this study contributes to provide useful information related to fenugreek genes up-regulated by the MeJA elicitation. Based on the phylogenetic tree construction and co-expression analysis with several known upstream genes, a set of specific CYP- and UGT- encoding genes have been proposed for involvement in the downstream steps from cholesterol to diosgenin (Scheme 1). Transcription factors (TFs) that might play regulatory effects on diosgenin biosynthesis were also discussed. Thus, this study may provide a strong basis for characterizing the downstream pathway steps up to disogenin, in turn pushing forward to the production of this important chemical by a synthetic biology approach.

## 2. Results and Discussion

### 2.1. Diosgenin Biosynthesis in Fenugreek was Improved by MeJA Stimuli

**Figure 1:**
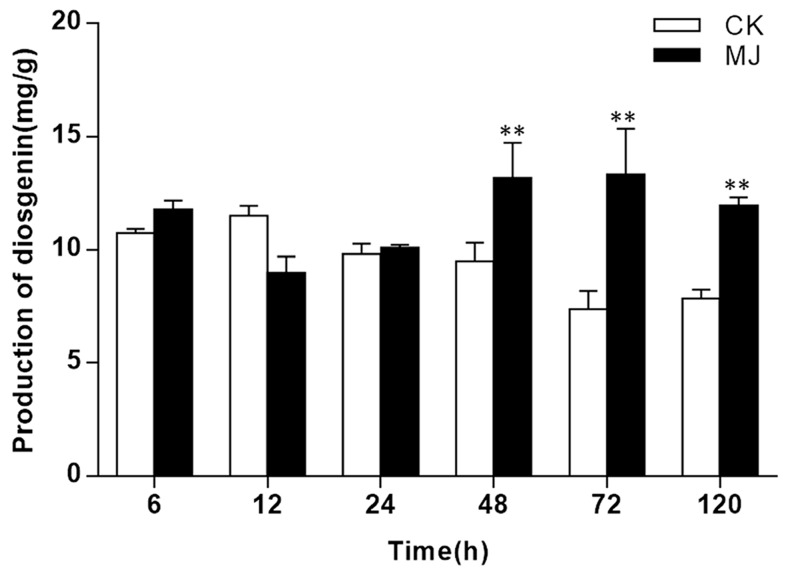
Comparison of diosgenin content between the MeJA-treated and control *T. foenum-graecum* seedlings under different time points. Error bars indicate ±SD of three biological replicates and tested by paired Student’s *t* test. The mark “**” indicate very significant difference (*p* < 0.01) relative to the corresponding controls at each time point. CK: the 24 h control samples. MJ: the 24 h MeJA-treated samples.

Compared to the controls, the MeJA treatment did not cause significant changes in diosgenin biosynthesis in the 6 h-, 12 h- and 24 h-treated plants with only a slight increase for the 6 h-treatment and a slight decrease for the 12 h-treatment (Figure 1), while at the 48 h-, 72 h- and 120 h-post treatment points, diosgenin contents in these MeJA-treated plants were significantly higher than those in their respective controls, showing improvements by 38.74%, 80.51% and 52.04%, respectively (Figure 1). This data suggested that MeJA could be an effective inducer for improving diosgenin biosynthesis in fenugreek and this improvement would start after a certain time point probably depending on the concentration of MeJA used.

### 2.2. De Novo Assembly and Functional Annotation

To produce sufficient sequencing data, the MeJA-treated seedlings under all the time points (6, 12, 24, 48, 72 and 120 h after treatment) were subjected to a RNA-sequencing analysis. All raw sequencing data have been deposited in NCBI database with the accession numbers of SRR8281654- SRR8281660. To identify the MeJA-induced genes, the 24 h-control lines were sequenced as well, and the MeJA-induced genes were identified by comparing the 24 h-MeJA-treated and the 24 h-control samples. After removing adapter and low quality sequences, a total of 389,479,556 clean reads were obtained with the values of Q20, Q30 and GC content shown in Table 1.

Using the Trinity software, the clean reads were applied to a de novo assembly analysis, which finally led to the construction of a total of 239,545 transcripts with an average length of 1036 bp. The minimum and maximum lengths of the transcripts were 200 bp and 17,640 bp, respectively, and the N50 length was 1766 bp. The functional annotation of the transcripts were conducted by sequence blasting analysis against several public databases including Nr, Nt, KOG/COG, Swiss-Prot, KO (KEGG Ortholog), Pfam, and GO databases. A total of 172,825 unigenes were successfully annotated (Table 2), of which 74.3% of the unigenes had the most hits from *Medicago sativa*, followed by *Cicer arietinum* (10.8%), *Glycine max* (2.6%), *Glycine soja* (1.6%), *Phaseolus vulgaris* (1.2%) and other speices (9.5%).

### 2.3. Gene Ontology (GO) Annotation

**Figure 2:**
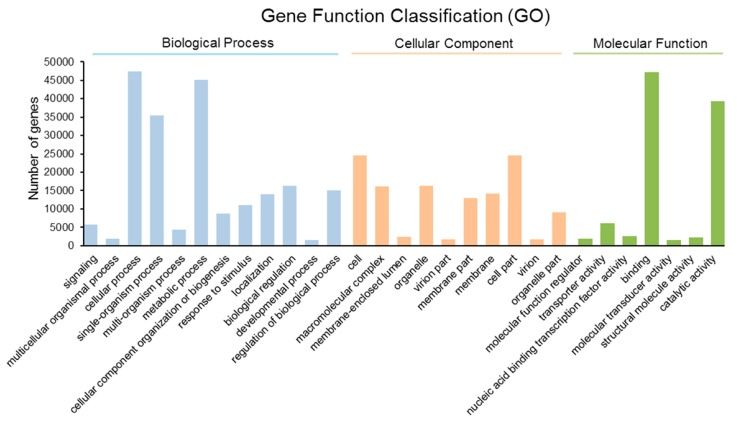
Gene ontology classification of the *T. foenum-graecum* unigenes.

Based on NR and Pfam annotations, a total of 83,000 unigenes were successfully assigned to the GO term annotation and then classified into three main categories: biological processes, cellular components, and molecular function. A majority of the unigenes were assigned to biological processes, followed by cellular components and molecular functions. The most common GO terms within biological processes included cellular process (47,458, GO: 0009987), metabolic process (45,050, GO: 0008152), single-organism process (35,541, GO: 0044699), biological regulation (16,351, GO: 0065007) and regulation of biological process (15,068, GO: 0050789). In cellular components class, the top three largest categories were cell (24,553, GO: 0005623), cell part (24,553, GO: 0044464), and organelle (16,375, GO: 0043226). In the molecular function category, the classes related to binding (47,261, GO: 0005488), catalytic activity (39,306, GO: 0003824), transporter activity (6071, GO: 0005215) were the most frequently observed (Figure 2). GO enrichment analysis was performed with the differentially expressed genes induced by the MeJA treatment at the 24 h time point. The “oxidation-reduction process” (GO: 0055114) represented the mostly enriched section in the category of “biological process” (Figure S1) while the highest enrichment is the “glutamate-cysteine ligase complex” (GO: 0017109) for the category “cellular component” (Figure S2) and the “oxidoreductase activity, acting on single donors with incorporation of molecular oxygen, incorporation of two atoms of oxygen” (GO: 0016702) for the category of “molecular function” (Figure S3).

### 2.4. Metabolic Pathway Analysis by KEGG

KEGG classification of the annotated unigenes was performed using the Kyoto Encyclopedia of Genes and Genomes (KEGG) automatic annotation server (KAAS). 

**Figure 3:**
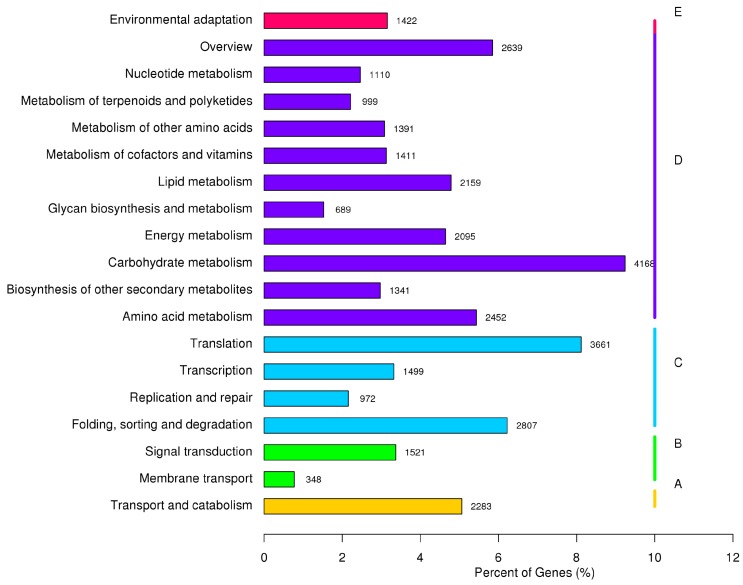
A KEGG classification of the *T. foenum-graecum* unigenes (**A**) Cellular Processes; (**B**) Environmental Information Processing; (**C**) Genetic Information Processing; (**D**) Metabolism, and (**E**), Organism Systems.

A total of 45,112 unigenes could be mapped to the KEGG database and the unigenes involved in the metabolic pathways of KEGG were divided into five groups: cellular processes, environmental information processing, genetic information processing, metabolism, and organism systems. 20,454 unigenes were grouped into a “metabolism” category and of which 4168 unigenes related to carbohydrate metabolism. 2283 and 1422 of the unigenes were clustered into “cellular processes” and “organismal systems”, respectively. The “environmental information processing” category included “membrane transport” (348) and “signal transduction” (1521). There were 8939 unigenes that were grouped into “genetic information processing” category and the majority of these unigenes relate to “translation” (3661) (Figure 3).

### 2.5. Identification of Genes Related to Steroidal Backbone Biosynthesis

Isopentenyl diphosphate (IPP) and dimethylallyl diphosphate (DMAPP) are two common blocks for the synthesis of steroidal compounds, to which diosgenin belongs. They are derived either from the MVA or from the MEP pathway. From the *T. foenum-graecum* transcriptome, a total of 34 unigenes, which putatively code for 14 enzymes in the MVA or MEP pathway, were discovered (Table 3). 

Based on the differential expression analysis, nearly all the MVA unigenes (AACT, HMGS, HMGR, MK, PMK, MDC) were up-regulated in response to the MeJA treatment, in contrast the MEP pathway genes (DXS, DXR, MCT, CMK, MECPS, HDS, HDR) in the 24 h MeJA-treated plants showed relatively lower or similar expression levels in comparison with those in the 24 h control samples. This data demonstrated that diosgenin biosynthesis might be primarily originated from the MVA pathway while not from the MEP origin. In the MVA pathway, HMG-CoA reductase (HMGR) is well considered as a rate-limiting enzyme [25], three HMGR-encoding unigenes (cluster-2140.36411, cluster-2140.78950 and cluster-2140.104061) were found in the fenugreek transcriptome. They were all induced by MeJA with their expression levels being improved by 2.65–372.56 folds (Table S1), confirming that the MVA pathway was indeed boosted by the MeJA treatment.

Cholesterol represents the backbone of diosgenin molecule, and in plants there are nine enzymes in the steps from cycloartenol to cholesterol [13,18] (Scheme 1). In the fenugreek transcriptome of this study, a total of 29 unigenes, including five for SSR, eight for SMO, two for CPI, six for CYP51, four for C14-R, one for 8,7 SI, two for C5-SD and one for 7-DR, were discovered in the steps from cycloartenol to cholesterol. Most of these cholesterol pathway unigenes were slightly or obviously up-regulated by the MeJA treatment at the 24 h time point (Table 3, Table S1). Taken together, the unigenes related to diosgenin backbone synthesis in *T. foenum-graecum* were generally up-regulated by the MeJA treatment.

### 2.6. Identification of the CYP- and UGT-encoding Genes in the Downstream Diosgenin Pathway

**Figure 4:**
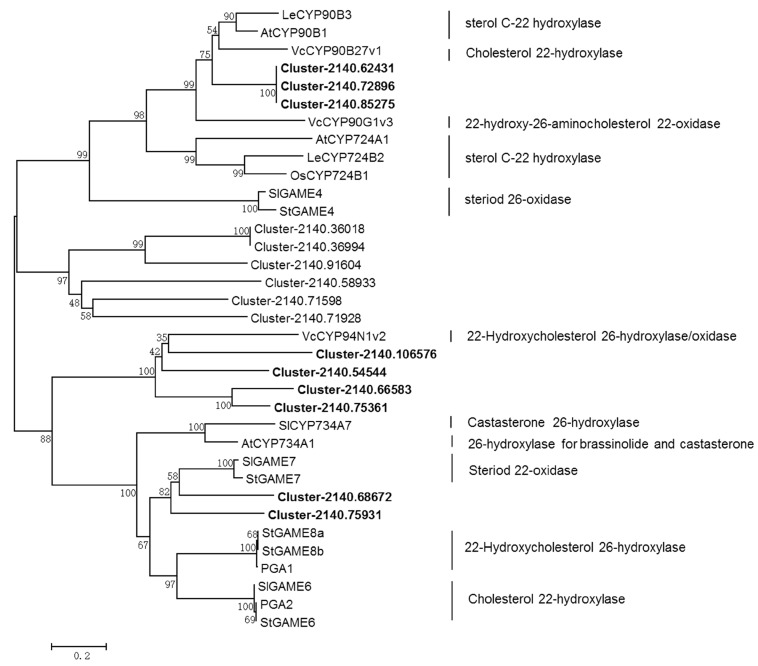
Phylogenetic tree analysis of the 15 MeJA-upregulated *T. foenum-graecum* CYP450 candidates with known steroid oxidizing CYP450s.

The genes involved in the downstream steps from cholesterol to diosgenin are largely unknown. Several reports proposed that multiple oxidizations at the C-22, C-16 and C-26 positions of cholesterol lead to the biosynthesis of diosgenin (Scheme 1), and these oxidizing steps were believed to be catalyzed by specific cytochrome P450 enzymes [13,26]. Based on the results mentioned above that the diosgenin backbone genes were generally induced by MeJA (Table S1), it is reasonable to speculate that the downstream P450 candidates involved in diosgenin biosynthesis might be induced by MeJA as well. Differential expression analysis led to the identification of 15 full-length P450 unigenes that were significantly up-regulated in response to the MeJA stimuli (Table S2). They belong to seven different subfamilies of CYP90, CYP86, CYP71, CYP736, CYP94, CYP93 and CYP72. Phylogenetic analysis showed that the P450 candidates of cluster-2140.62431, cluster-2140.72896 and cluster-85275 displayed a closer relationship to steroid C-22 hydroxylases that included AtCYP90B1 from *Arabidopsis thaliana* [27], LeCYP90B3 from *Lycopersicon esculentum* [28] and VcCYP90B27v1 from *Veratrum californicum* [29] (Figure 4). This data suggested that cluster-2140.62431, cluster-2140.72896 and cluster-85275 are the potential candidates catalyzing the C-22 hydroxylation reaction during diosgenin biosynthesis. The candidates of cluster-2140.75931 and cluster-2140.68672, which belong to the subfamily of CYP72, showed a relatively higher homology to StGAME7 from *Solanum tuberosum* [30] which function is not known yet but was ever indicative of playing a role in the E ring closure for steroid compounds, suggesting that cluster-2140.75931 or cluster-2140.68672 might be involved in the E ring formation for diosgenin biosynthesis. On the other hand, four unigenes (cluster-2140.106576, cluster-2140.54544, cluster-2140.66583 and cluster-2140.75361) had high sequence similarities to VcCYP94N1v2 which had been proved to be a 22-hydroxycholesterol 26-hydroxylase/oxidase [29]. Thus, these four candidates belonging to CYP94 family are likely to be the CYP enzyme for catalyzing the C-26 oxidation in diosgenin biosynthesis.

So far, no CYP enzymes have been found to catalyze the C-16 oxidation in steroid biosynthesis, however a 2-oxoglutarate-dependent dioxygenase (St16DOX/Sl16DOX) was recently reported to catalyze a C16-hydroxylation for SGA biosynthesis from *Solanum tuberosum* or *Solanum lycopersicum* [31]. From the fenugreek transcriptome of this study, nine unigenes, which included cluster-10627.0, cluster-10584.1, cluster-2140.72521, cluster-2140.14034, cluster-2140.135954, cluster-2140.14222, cluster-2140.122743, cluster-2140.11716 and cluster-10584.0, shared above 40% amino sequence identity to St16DOX/Sl16DOX. Among these nine candidates, only cluster-2140.72521 and cluster-2140.14034 were induced by the MeJA treatment while the rest candidates were not.

In addition to CYP enzymes, a specific C-26 glucosyltransferase and a 26-*O*-β-glucosidase are proposed to catalyze the reactions which facilitate the closure of diosgenin ring E and ring F (Scheme 1) [20,26,32]. Within the UGT unigenes with a length above 1000 bps in the *T. foenum-graecum* transcriptome, there were 21 UGT unigenes that were significantly induced by the MeJA elicitation (Table S3). Because that until now the C-26-*O*-glucosyltransferase has never been reported from any plant species, it is difficult to predict the C-26-UGT candidates by a phylogenetic tree analysis (Figure S4). On the other hand, there were 11 glucosidase annotated unigenes with a length above 1000 bps that were induced by the MeJA elicitation (Table S4). A phylogenetic analysis showed that the candidates of cluster-2140.60643 and cluster-2140.23403 showed a relatively closer relationship with the isoflavone β-glucosidase from *Glycine max* [33] and the furostanol glycoside 26-*O*-β-glucosidases from *Dioscorea esculenta* [20] and *Costus speciosus* [19] (Figure S5), suggesting that they may be the C-26-β-glucosidase candidates during diosgenin biosynthesis.

In short, by a comparatively transcriptome analysis, this study provided the candidate genes that might be involved in the downstream steps from cholesterol to diosgenin. However, the full understanding of the role of these candidates may be achieved if knock down approaches were performed in fenugreek itself or their biochemical functions were studied in microorganisms by a synthetic biology approach.

### 2.7. Co-expression Analysis of the MeJA-induced CYP- and UGT-encoding Genes with the HMGR and SS

**Figure 5:**
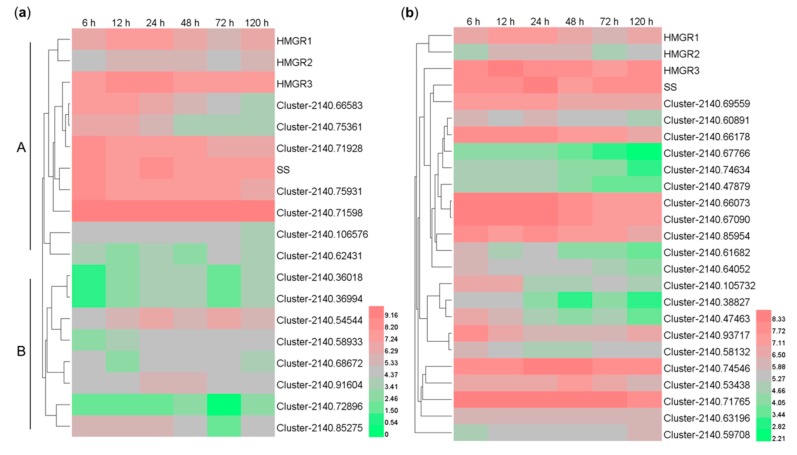
Gene cluster analysis of the MeJA-induced CYP450 (**a**) and UGT (**b**) unigenes with the HMGR- and SS-encoding genes across the different MeJA-treating time points.

HMGR and SS represent the rate-limiting or regulatory backbone enzymes for saponin biosynthesis [34]. In the *T. foenum-graecum* transcriptome, there were three unigenes coding for HMGR (HMGR1, cluster-2140.36411; HMGR2, cluster-2140.104061; HMGR3, cluster-2140.78950) and one unigene for SS (cluster-2140.75139). These backbone genes, together with the MeJA-induced CYP- and GT-encoding unigenes, were used for gene cluster analysis across all the time points (6, 12, 24, 48, 72 and 120 h) after MeJA treatment. Based on the gene expression patterns shown in Figure 5a, the MeJA-induced CYP unigenes could be divided into two clusters (A and B). CYP members of cluster A showed relatively closer expression patterns with the backbone unigenes (HMGR1, HMGR2, HMGR3 and SS), while members from the cluster B displayed distinctly different patterns. This data suggested that the CYP members from the cluster A are most likely involved in diosgenin biosynthesis. Interestingly, several CYP members from the cluster A (cluster-2140.66583, cluster-2140.75361, cluster-2140.75931, cluster-2140.106576, and cluster-2140.62431) were also suggested to be the CYP candidates in diosgenin biosynthesis by the phylogenetic tree analysis (Figure 4). For the UGT unigenes, the expression trends of cluster-2140.69559, was consistent with the SS, while the expression patterns of the other UGT unigenes showed poor similarities with those of the backbone genes (Figure 5b).

### 2.8. Identification of the MeJA-induced Transcription Factors

A total of 4499 unigenes were annotated as transcription factors (TFs), and they were grouped into different families such as WRKY, CPP, NF-YB, bHLH and AP2/ERF. It has been reported that members of WRKY, bHLH and AP2/ERF participate in regulating the biosynthesis of terpenoids, alkaloids and steroids [35,36,37]. Out of 4499 unigenes annotated as TFs, 20 TF unigenes showed obviously increased expressions in response to the MeJA treatment (Table S5). These MeJA up-regulated TFs belong to families of NAC, WRKY, bHLH, TIFY, HD-ZIP, AP2/ERF and BTB/POZ, of which members of WRKY, bHLH and TIFY were the most abundant. They constitute a valuable gene resource for the further studies of their regulatory functions in diosgenin biosynthesis.

### 2.9. Validation of Selected Transcripts by qRT-PCR

**Figure 6:**
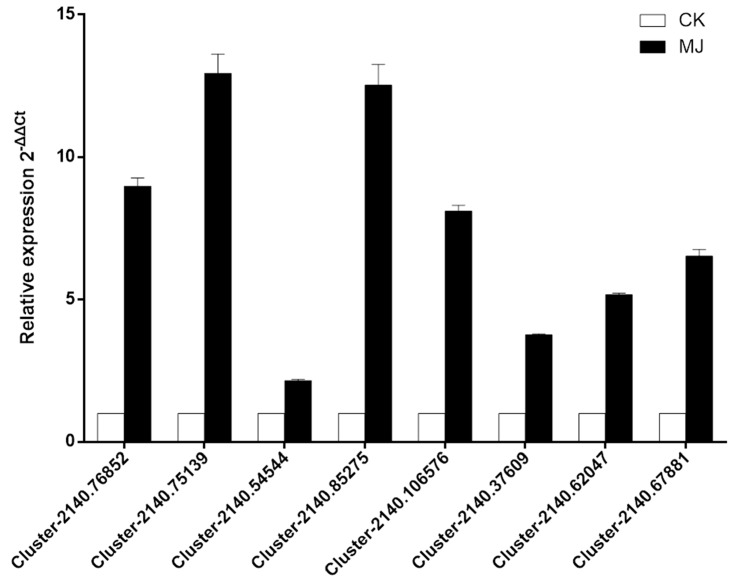
Real-time PCR analysis of several candidate unigenes between the 24 h MeJA-treated and the 24 h control samples. The candidate unigenes included one coding for HMGS (cluster-2140.76852), one for SS (cluster-2140.75139), three for CYPs (cluster-2140.54544, cluster-2140.85275 and cluster-2140.106576), and three for TFs (cluster-2140.37609, cluster-2140.62047, and cluster-2140.67881). CK: the 24 h control samples. MJ: the 24 h MeJA-treated samples.

In order to confirm the reliability of the gene expression data by the RNA-sequencing technique, real-time PCRs were used to compare the expression of several potential candidates between the MeJA 24 h-treated samples and the 24 h-control lines. The selected candidates included one coding for HMGS (cluster-2140.76852), one for SS (cluster-2140.75139), three for CYPs (cluster-2140.54544, cluster-2140.85275 and cluster-2140.106576), and three for TFs (cluster-2140.37609, cluster-2140.62047, and cluster-2140.67881). As shown in Figure 6, the gene expression trend of the qRT-PCR data matched very well with that retired from the RNA-sequencing data (Table S6), suggesting that the differential expression data by the sequencing is reliable.

## 3. Materials and Methods

### 3.1. Plant Material

*T. foenum-graecum* seeds from Shanxi Province in China were used in this study. The seeds were sterilized by a one minute-immersion in 70% ethanol and a ten minute-immersion in sodium hypochlorite 5% (*w*/*v*) followed by three times of distilled water washes, respectively. The sterile seeds were incubated in water-agar medium and germinated in the dark at 25 ± 2 °C for two days.

### 3.2. MeJA Treatment

MeJA (Sigma Aldrich, St. Louis, MO, USA) was dissolved in absolute ethanol and diluted into 1/2 MS liquid media at a final concentration of 0.015% (*v*/*v*) to get the MeJA solution. The same concentration of ethanol was used as the mock control. For the treatments, the germinated seedlings with similar growth sizes were incubated in the MeJA or the mock ethanol solution where sterilized glass beads were used to fix the seedlings. The treated seedlings were grown in a chamber at 25 ± 2 °C with a 16 h light photoperiod and were harvested at 6, 12, 24, 48, 72 and 120 h after the treatment. For each treatment, three biological replicates were performed. The collected seedlings were divided into two parts: the samples of part one were immediately frozen in liquid nitrogen and stored at −80 °C for the RNA-sequencing analysis and real-time PCR analysis, the samples of part two were kept in oven at 45 °C till complete dryness for diosgenin extraction.

### 3.3. Diosgenin Extraction and Analysis

Dried fenugreek seedlings collected at each time point were ground to fine powder, and 0.02 g of accurately weighted powder was extracted for 30 min with 1 mL methanol under sonication (180 W, 40 KHz). The extraction was repeated for three times and the clear extracts were combined, evaporated to dryness and the residues were hydrolyzed with 2 mL 1.5 M sulfuric acid for 4 h at 100 °C. After the acid hydrolysis, the residue was extracted with 2 mL *n*-hexane for three times and subsequently washed with 2 mL 1% NaHCO3 and 2 mL distilled water. Organic phase was collected, evaporated to dryness on vacuum and re-dissolved in 500 μL mobile phase (acetonitrile: water 90:10) and filtered through 0.22 µm filter prior to high performance liquid chromatography (HPLC) analysis. As an internal standard, 60 µg of ursolic acid was added to the samples before the extraction. HPLC analysis was performed on an LC-20AT instrument (Shimadzu, Kyoto, Japan) using an Inertsil ODS-SP reverse phase column (250 mm × 4.6 mm, 5 µm). The flow rate was 1.0 mL/min and the column temperature was set at 30 °C. A solution of 88% methanol and 12% phosphoric acid water (0.01%, *v*/*v*) was used as the mobile phase. The monitoring wave length was set to 204 nm.

### 3.4. RNA Extraction and Sequencing

Total RNAs were extracted from the collected samples using an EASYspin Plus Kit according to the manufacturer’s instructions (Aidlab Biotechnologies Co. Ltd., Beijing, China). The quality and quantity of the extracted RNA were monitored by an agar gel electrophoresis and a BioPhotometer (Eppendorf, Hamburg, Germany) analysis. The total RNA of three biological repeats was mixed in equal amounts into a single pool for RNA-Seq. Library construction and sequencing were performed at Novogene Company (Beijing, China). To construct the cDNA library, mRNAs were purified and sheared into short fragments which served as templates for synthesizing the cDNAs using random hexamer primers. After being connected with the sequencing adapters, the cDNAs were purified and then were subjected to the sequencing on an the HiSeq2000 platform (Illumina, San Diego, CA, USA). Clean reads were obtained by removing adapter sequences, low quality reads (more than 50% bases with Qphred value ≤ 20) and reads containing high percentage of N (>10%).

### 3.5. Digital Differential Gene Expression Analysis

The Trinity software was employed to assemble the transcripts. To determine the expression of each transcript, clean reads were mapped individually to the assembled transcriptome, and the transcript abundance of each unigene was normalized using the FPKM (fragments per kilobase per million mapped reads) method [38]. Differentially gene expression analysis was performed using DEGseq R package with a threshold of |log2 (fold change)| > 1 and corrected q.value < 0.005 [39,40].

### 3.6. Validation of transcripts by Quantitative Real-Time PCR (qRT-PCR)

Quantitative expression of genes putatively involved in diosgenin biosynthesis was analyzed using qRT-PCRs. The primers used in the qRT-PCRs are shown in Table S6. The qRT-PCR reaction was performed on an ABI 7500 Real-Time PCR System using the FastStart Universal SYBR Green Master Mix (Roche, Mannheim, Germany) using the following program: 95 °C for 10 min, followed by 40 cycles of 95 °C for 15 s, and 60 °C for 60 s. The fenugreek actin gene (unigene cluster-2140.65830) was selected as an internal reference gene for the normalization and three technical replicates were performed for each sample. Relative expression levels for each unigene were calculated using 2^−ΔΔ*C*t^ method.

### 3.7. Gene Cluster Analysis

RNA-seq transcriptome data from all the time points (6, 12, 24, 48, 72 and 120 h) after MeJA treatment was obtained for clustering analysis. Clustering of the MeJA-induced CYP450 and UGT unigenes with the backbone genes HMGR (HMGR1, cluster-2140.36411; HMGR2, cluster-2140.104061; HMGR3, cluster-2140.78950) and SS (cluster-2140.75139) was performed using Heml 1.0 (Heatmap Illustrator, Wuhan, China) [41]. The normalization of the transcript abundance of these unigenes was performed by the logarithm method using the FPKM values. Average linkage hierarchical clustering and row-wise normalization were used to present the heat maps.

## Figures and Tables

**Table 1:** Statistic of sequencing and de novo assembling of the *T. foenum-graecum* transcriptome.

	Raw Reads	Clean Reads	Q20 (%)	Q30 (%)	GC (%)
MJ6	68803276	67560716	97.54	93.60	41.63
MJ12	48826160	47953056	97.62	93.77	41.98
MJ24	50687102	49648026	97.72	94.02	41.71
MJ48	56366906	55413478	97.55	93.65	41.85
MJ72	56903406	55842268	97.60	93.78	41.88
MJ120	62961696	61951984	97.50	93.59	41.93
CK24	52183650	51110028	97.43	93.47	42.06

**Table 2:** Summary of annotations of the *T. foenum-graecum* unigenes.

Annotation	Number of Genes	Percentage (%)
Annotated in NR	118,631	68.64
Annotated in NT	137,541	79.58
Annotated in KO	45,112	26.10
Annotated in Swiss-Prot	89,801	51.96
Annotated in Pfam	82,876	47.95
Annotated in GO	83,000	48.02
Annotated in KOG	30,687	17.75
Total Unigenes	172,825	100

**Table 3:** Putative genes involved in cholesterol biosynthesis in *T. foenumgraecum*.

Gene Name	EC Number	Gene ID	Total Unigenes
AACT	2.3.1.9	Cluster-2140.52353, 2140.68129, 2140.31592,2140.74635, 2140.75440, 2140.31592, 2140.63638	7
HMGS	2.3.3.10	Cluster-2140.76852, 2140.76851, 2140.124037	3
HMGR	1.1.1.34	Cluster-2140.36411, 2140.78950, 2140.104061	3
MK	2.7.1.36	Cluster-2140.53808, 2140.53807	2
PMK	2.7.4.2	Cluster-2140.37776	1
MDC	4.1.1.33	Cluster-2140.84671	1
DXS	2.2.1.7	Cluster-2140.115496, 2140.69916, 2140.52884, 2140.108949, 2140.69095	5
DXR	1.1.1.267	Cluster-2140.80427	1
MCT	2.7.7.60	Cluster-2140.48880, 2140.39394, 2140.69765	3
CMK	2.7.1.148	Cluster-2140.108677	1
MECPS	4.6.1.12	Cluster-2140.49224, 2140.56745	2
HDS	1.17.7.1	Cluster-2140.79758, 2140.66344	2
HDR	1.17.1.4	Cluster-2140.76895	1
IDI	5.3.3.2	Cluster-2140.68527, 2140.68885	2
GPPS	2.5.1.29	Cluster-2140.83026	1
FPPS	2.5.1.10	Cluster-2140.77163	1
SS	2.5.1.21	Cluster-2140.75139	1
SE	1.14.14.17	Cluster-2140.68704, 2140.63913, 2140.63916, 2140.55508, 2140.65244, 2140.63914	6
CAS	5.4.99.8	Cluster-2140.68038, 2140.75982, 2140.67914, 2140.68038	3
SSR	1.3.1.72	Cluster-2140.73628, 2140.75526, 2140.71445, 2140.65744, 2140.65030	5
SMO	1.14.13.72	Cluster-2140.88745, 2140.50156, 2140.82687, 2140.51993, 2140.62857, 2140.62855, 2140.43204, 2140.62858	8
CPI	5.5.1.9	Cluster-2140.77401, 2140.52812	2
CYP51	1.14.13.70	Cluster-2140.73183, 2140.70550, 2140.65854, 2140.71941, 2140.84199, 2140.65644	6
C14-R	1.3.1.70	Cluster-2140.69242, 2140.69243, 2140.69244, 2140.56203	4
8,7 SI	5.3.3.5	Cluster-2140.71662	1
C5-SD	1.14.19.20	Cluster-2140.68714, 2140.72000	2
7-DR	1.3.1.21	Cluster-2140.70707	1

## Supplementary Materials

The following are available online. Table S1: Transcript abundance (FPKM values) of the backbone unigenes for cholesterol biosynthesis under all the MeJA-treated time points. The expression levels of the candidate genes in the 24 h-MeJA and the 24 h-control lines were highlighted by red colors. Table S2: Transcript abundance (FPKM values) of the potential CYP candidates under all the MeJA-treated time points. The expression levels of the CYP candidate genes in the 24 h-MeJA and the 24 h-control lines were highlighted by red colors. Table S3: Transcript abundance (FPKM values) of the UGT candidates under all the MeJA-treated time points. The expression levels of the UGT candidate genes in the 24 h-MeJA and the 24 h-control lines were highlighted by red colors. Table S4: Transcript abundance (FPKM values) of the β-glucosidase candidates under all the MeJA-treated time points. The expression levels of the β-glucosidase candidate genes in the 24 h-MeJA and the 24 h-control lines were highlighted by red colors. Table S5: Transcript abundance (FPKM values) of the transcription factor (TF) candidates under all the MeJA-treated time points. The expression levels of the TF candidate genes in the 24 h-MeJA and the 24 h-control lines were highlighted by red colors. Table S6: Primers for amplifying transcripts in the qRT-PCR experiment. Figure S1: GO enrichment analysis for the MeJA-induced differentially expressed genes in the category of “biological process” Figure S2: GO enrichment analysis for the MeJA-induced differentially expressed genes in the category of “cellular component” Figure S3: GO enrichment analysis for the MeJA-induced differentially expressed genes in the category of “molecular function”. Figure S4: Phylogenetic tree analysis of the MeJA-upregulated *T. foenum-graecum* UGT candidates with known UGTs. Figure S5: Phylogenetic tree analysis of the MeJA-upregulated *T. foenum-graecum* β-glucosidase candidates with known β-glucosidases.
