# Supplementary material for: Comparative Transcriptome Analysis Identifies Genes Involved in Diosgenin Biosynthesis in Trigonella foenum-graecum L
Source: Molecules. 2019 Jan 1;24(1):140. doi: 10.3390/molecules24010140 (PMC6337231; doi:10.3390/molecules24010140)
Supplement: Supplementary file 1 [file molecules-24-00140-s001.zip › supplementary files/Table S6.docx]

Table S6. Primers for amplifying transcripts in qRT-PCRs.

| No | Name | Sequence (5’-3’) | Gene ID |
| --- | --- | --- | --- |
| 1 | HMGS-F | AGTTTTGCTCGTTTGGTCTTC | Cluster-2140.76852 |
| 2 | HMGS-R | GGTTGTACCTTCTCATCGTAT |  |
| 3 | SS-F | CCCCTCTAATTTTCGCTTTTG | Cluster-2140.75139 |
| 4 | SS-R | TCGCCTCATTTTAACTACCCC |  |
| 5 | P1-F | TTTGTGGCTTGGCTTTCGGT | Cluster-2140.54544 |
| 6 | P1-R | TTTAAGACTTCGGCTTAGGG |  |
| 7 | P2-F | ATGGTGTTGTTTCTGCTCCT | Cluster-2140.85275 |
| 8 | P2-R | TCCCTCTTGGTTTCTCTTGA |  |
| 9 | P3-F | GTTCCGTAGTTCTTGGATGTG | Cluster-2140.106576 |
| 10 | P3-R | ATTCTTTGCTTGCTTTGGTCA |  |
| 11 | U1-F | GGTTTAGGTTTCTTTCCTCTCTG | Cluster-2140.74546 |
| 12 | U1-R | AGTTGTGGTAATTGCATCTTTGT |  |
| 13 | U2-F | ATGGTGCTAGCTATCTTGCTCGT | Cluster-2140.85954 |
| 14 | U2-R | CGTCAACTCATCTGGTAAATCCG |  |
| 15 | U3-F | TGCTACCACCATCTATGCAGATA | Cluster-2140.66178 |
| 16 | U3-R | AGGCAACCCAGAAAGTTCAATTC |  |
| 17 | T1-F | GAACAACTTGACAACAAAGAACC | Cluster-2140.37609 |
| 18 | T1-R | GGAAAATCGTCGAATACAATAAC |  |
| 19 | T2-F | CTGCCCCTTCACCTGTTCCTA | Cluster-2140.62047 |
| 10 | T2-R | CCAATCTGTCTCGCCGCTTCT |  |
| 21 | T3-F | GAAACTACAAAGCCACAAGGAC | Cluster-2140.67881 |
| 22 | T3-R | GGAATAAGAGCAGCTAGAGCAA |  |
| 23 | T4-F | GTCAAAAAGAAGGTGCAAAGAAG | Cluster-2140.89728 |
| 24 | T4-R | GCTAAGAGACGCTGAACAAGGTA |  |
| 25 | Actin-tF | GGCGGGATGGTATTGAACTTTGTAC | -- |
| 26 | Actin-tR | ATCCTGAAACTCATCTTCCTCCTAA |  |
