# Supplementary figures and images for: Comparative Transcriptome Analysis Identifies Genes Involved in Diosgenin Biosynthesis in Trigonella foenum-graecum L
Source: Molecules. 2019 Jan 1;24(1):140. doi: 10.3390/molecules24010140 (PMC6337231; doi:10.3390/molecules24010140)

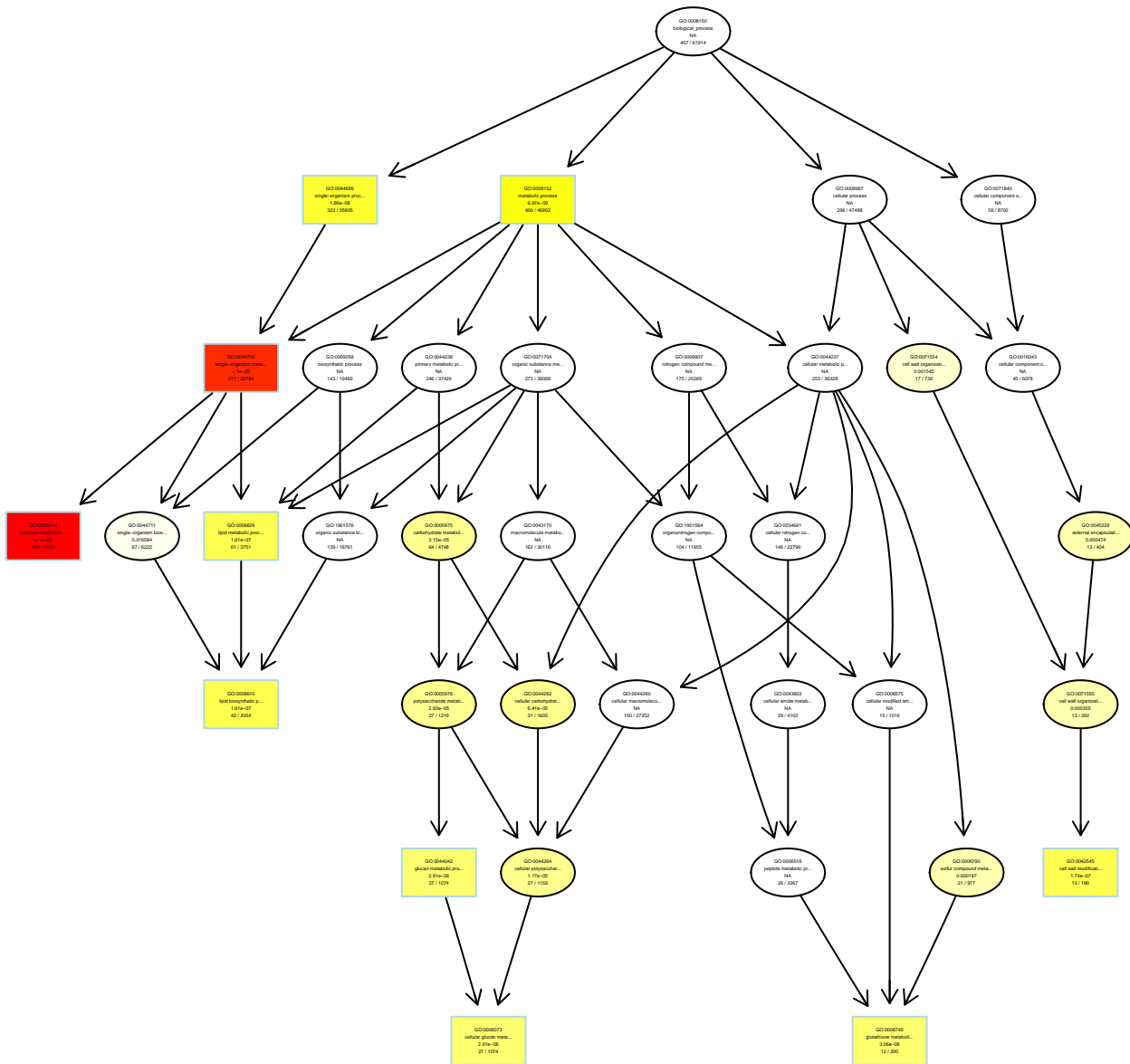

Supplement: Supplementary file 1 [file molecules-24-00140-s001.zip › supplementary files/Figure S1.pdf]

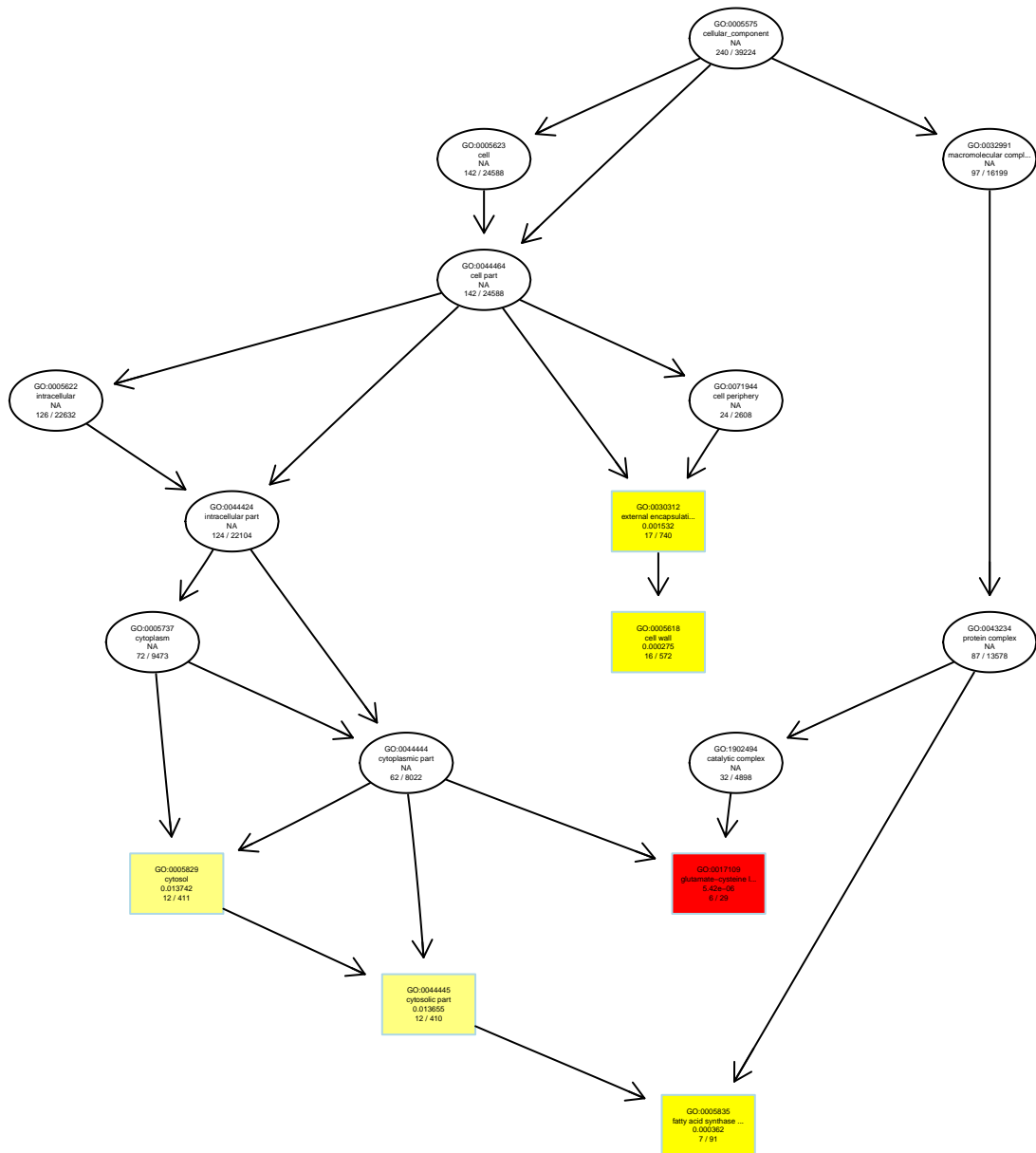

Supplement: Supplementary file 1 [file molecules-24-00140-s001.zip › supplementary files/Figure S2.pdf]

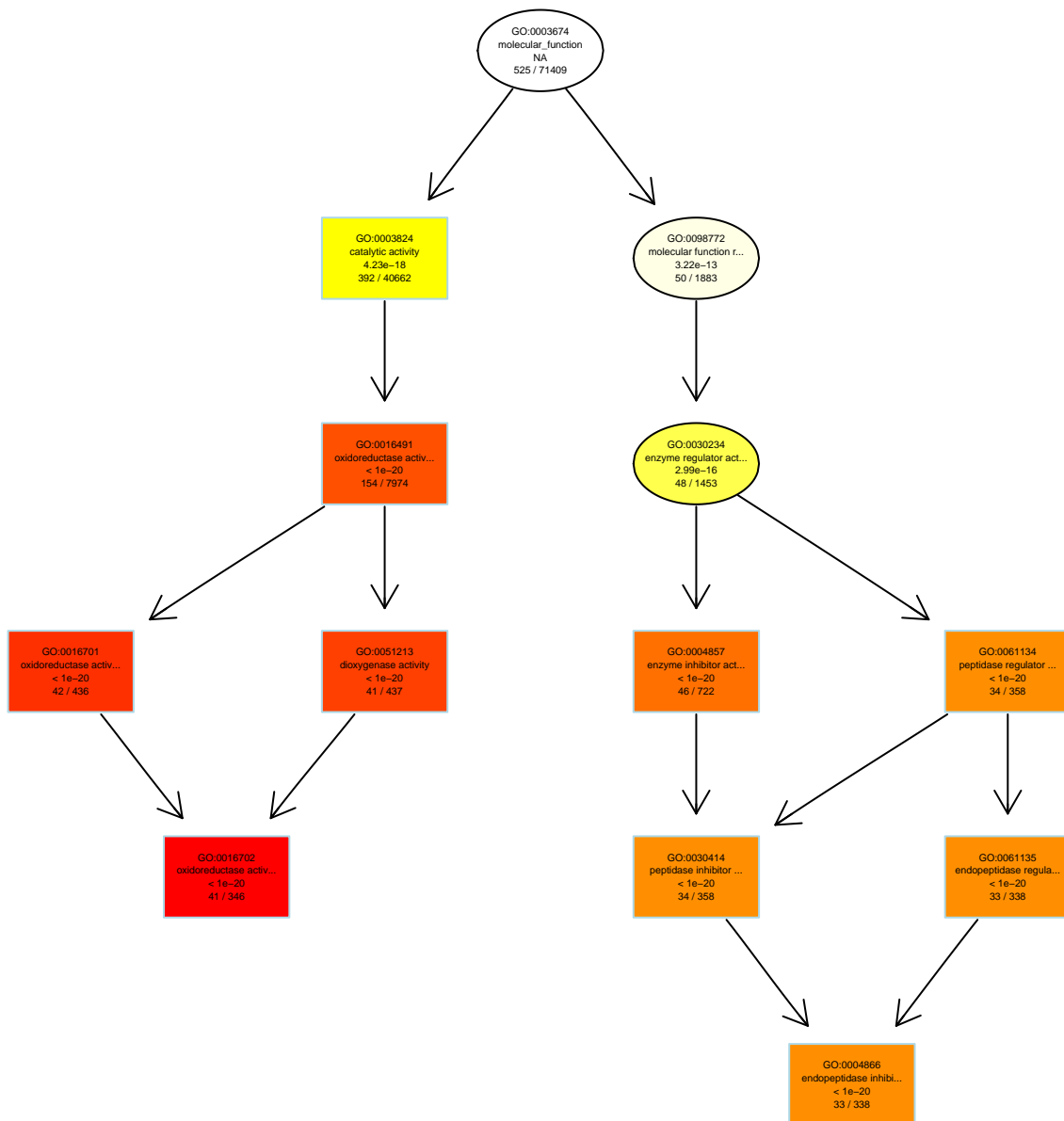

Supplement: Supplementary file 1 [file molecules-24-00140-s001.zip › supplementary files/Figure S3.pdf]

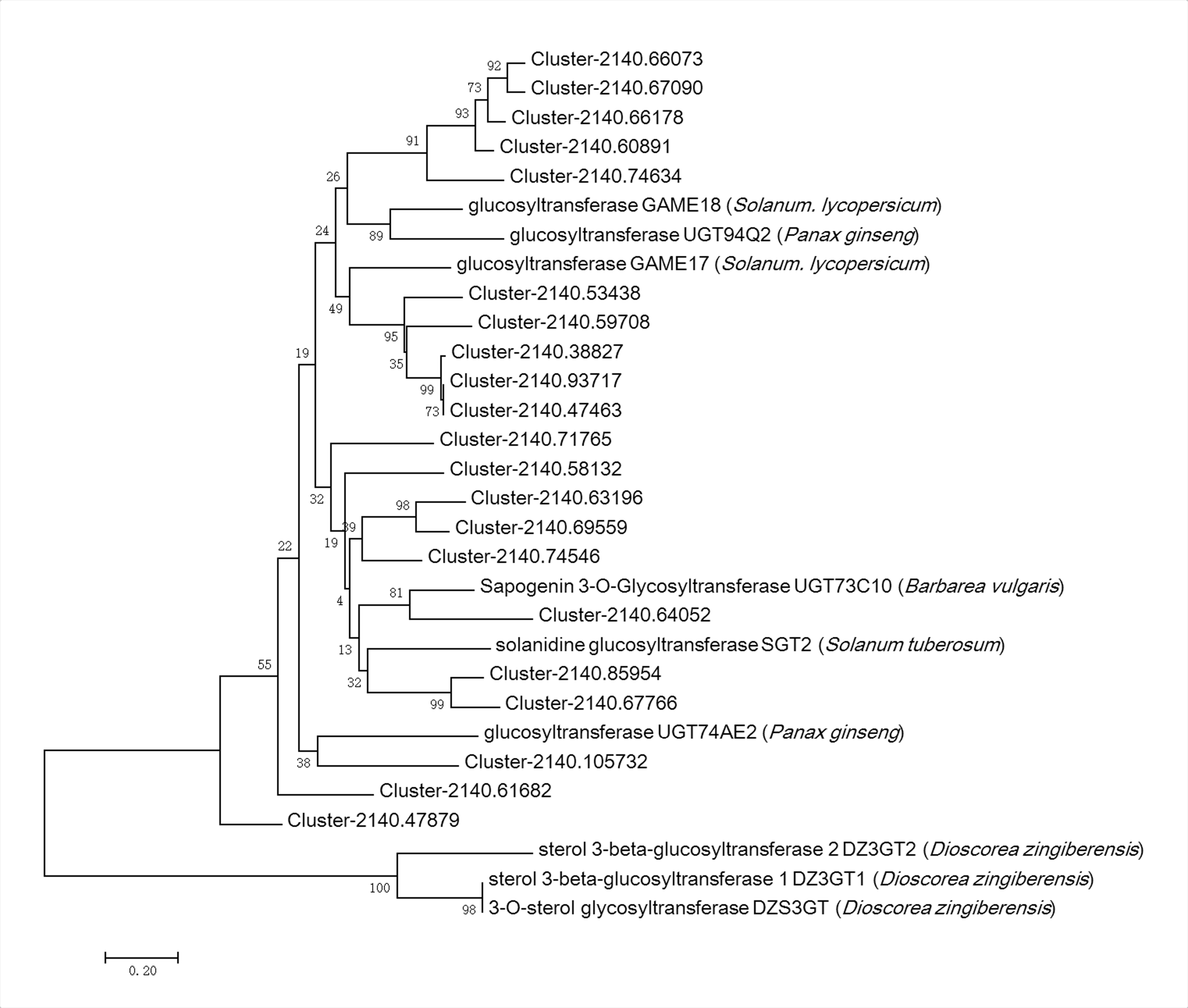

Supplement: Supplementary file 1 [file molecules-24-00140-s001.zip › supplementary files/Figure S4.tif]

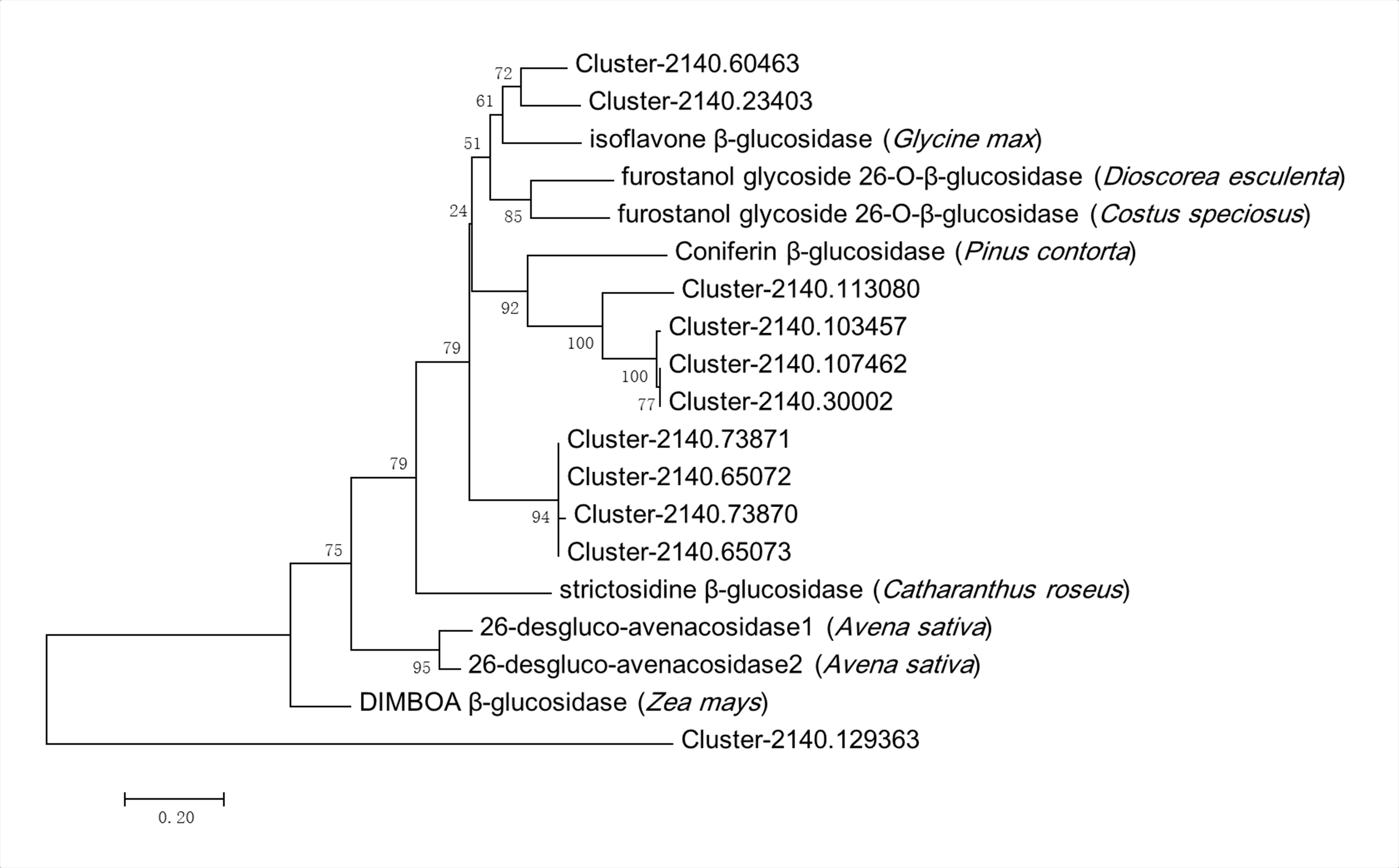

Supplement: Supplementary file 1 [file molecules-24-00140-s001.zip › supplementary files/Figure S5.tif]
